# Effects of Berberine on Cell Cycle, DNA, Reactive Oxygen Species, and Apoptosis in L929 Murine Fibroblast Cells

**DOI:** 10.1155/2015/796306

**Published:** 2015-10-13

**Authors:** Manman Gu, Jing Xu, Chunyang Han, Youxi Kang, Tengfei Liu, Yanfei He, Yanfei Huang, Cuiyan Liu

**Affiliations:** College of Animal Science and Technology, Anhui Agricultural University, 130 Changjiang West Road, Hefei, Anhui 230036, China

## Abstract

Berberine, an isoquinoline alkaloid isolated from several traditional Chinese herbal medicines (TCM), exhibits a strong antimicrobial activity in the treatment of diarrhea. However, it causes human as well as animal toxicity from heavy dosage. The present study was conducted to investigate the cytotoxicity of berberine and its possible trigger mechanisms resulting in cell cycle arrest, DNA damage, ROS (reactive oxygen species) level, mitochondrial membrane potential change, and cell apoptosis in L929 murine fibroblast (L929) cells. The cells were cultured *in vitro* and treated with different concentrations of berberine for 24 h. The results showed that cell viability was significantly decreased in a subjected dose-dependent state; berberine concentrations were higher than 0.05 mg/mL. Berberine at a concentration above 0.1 mg/mL altered the morphology of L929 cells. Cells at G2/M phase were clear that the level of ROS and cell apoptosis rates increased in 0.1 mg/mL group. Each DNA damage indicator score (DIS) increased in groups where concentration of berberine was above 0.025 mg/mL. The mitochondrial membrane potential counteractive balance mechanics were significantly altered when concentrations of berberine were above 0.005 mg/mL. In all, the present study suggested that berberine at high dosage exhibited cytotoxicity on L929 which was related to resultant: cell cycle arrest; DNA damage; accumulation of intracellular ROS; reduction of mitochondrial membrane potential; and cell apoptosis.

## 1. Introduction

**Figure 1:**
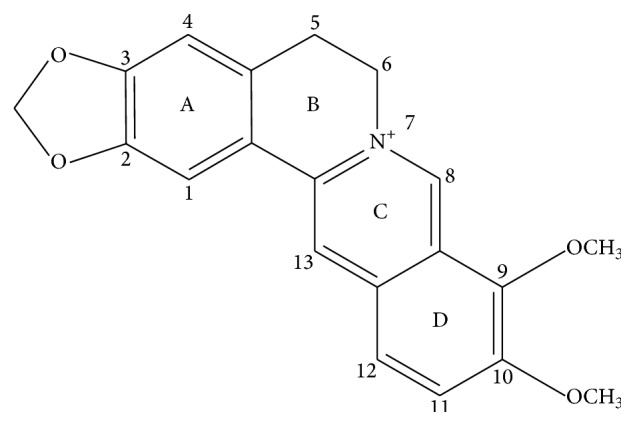
The molecular structure of berberine.

Berberine is an isoquinoline alkaloid isolated from several traditional Chinese herbal medicines (TCM) such as* Coptis chinensis*,* Berberis aristata*, and* Coptis japonica* [1] (Figure 1). As the most abundant alkaloid in* Rhizoma Coptidis*, berberine has been used extensively to treat diarrhea, clear heat, and remove toxicity in applied TCM practice. Multiple pharmacological observances, resulting from modern research of the effects of berberine, have demonstrated activities relating to efficacy of antioxidants [2], hepatoprotective effect [3, 4], lowering blood glucose [5–7], lipid-lowering [8], and antineoplastic [9, 10] and antiarrhythmic effect [11].

Since 1978, berberine was classed as a toxic substance in Singapore. This resulted from discoveries that dosage of berberine leads to the neonates suffering hemolytic jaundice or kernicterus. Since that time, subsequent research has been conducted to further investigate issues relating to toxicity resulting from berberine dosage. Almost all the studies have pointed out that adverse effects are resultant yet attributed to excessive dosage (more than 4 g) and improper diagnostic application of the substance [12]. There have been many more reports related to the toxicity of berberine* in vivo*, but it remains unclear that the toxicity and its relevant mechanisms are directly resultant of berberine specific dosage; particularly questioned are proposed findings of those at the cellular and genetic levels. In order to effectively evaluate berberine, a medicine that has broad development prospects, this study was carried out to explore the effects and the possible mechanisms of berberine on L929 mouse fibroblasts. Observance revealing the resultant effects and special focus on potential for management of toxicity levels provide a theoretical basis for secure application in practice and rationalize evaluation of the pharmacological effects of applicative use of berberine as a legitimate and secure treatment.

## 2. Materials and Methods

### 2.1. Chemicals and Agents

Berberine was purchased from Sigma Aldrich (St. Louis, MO, USA). In trials it was dissolved in dimethyl sulfoxide (DMSO) (DMSO final concentration < 0.5%) and then immediately diluted with the cell culture medium prior to use. Fetal bovine serum (FBS) was purchased from Life Technologies Co. (Shanghai, China) and high-glucose Dulbecco's Modified Eagle's Medium (DMEM) was obtained from Thermo Fisher Scientific Inc. (Beijing, China). Cell Counting Kit-8 (CCK-8) was acquired from Dojindo Laboratories (Shanghai, China). Cell cycle assay kit and Annexin V-FITC/PI Kit were obtained from Becton Dickinson (Franklin Lakes, New Jersey, USA). Reactive Oxygen Species Assay Kit and mitochondrial membrane potential kit with JC-1 were obtained from Beyotime Institute of Biotechnology (Shanghai, China). Low melting agarose (LMA) was supplied by Amresco Corporation (Shanghai, China). Phenol, dimethyl sulfoxide (DMSO), vinorelbine ditartrate salt hydrate (VNB), H_2_O_2_, and cisplatin were purchased from Sigma Aldrich (St. Louis, MO, USA). Gel-Green was purchased from Biotium Inc. (Investment Blvd., Hayward, US). All the other materials were of analytical grade, including NaCl, Na_2_-EDTA, Tris, Sodium N-lauroylsarcosine (SLS), NaOH, 1% Triton-100, Tris-base, HCl, Na_2_HPO_4_, KH_2_PO_4_, and KCl.

### 2.2. Cell Culture

L929 murine fibroblast (L929) cell line was purchased from the Culture Collection of the Chinese Academy of Sciences (Shanghai, China). Cells were cultured in DMEM supplemented with 10% FBS at 37°C in a saturated humidified atmosphere of 5% CO_2_, and when 85% confluence was achieved, L929 cells were dissociated using 0.25% trypsin and then channeled appropriately for observant experimental analyses.

### 2.3. Assessment of Cell Viability

L929 cell viability was determined by CCK-8 assay after treatment with different berberine. The positive control was treated with 0.64% phenol. Briefly, L929 cells were seeded in 96-well tissue culture plates (100 *μ*L/well) at a density of 8 × 10^4^ cells/well. After the cells formed a 60–70% confluent monolayer, the culture medium of each well was carefully refreshed by 100 *μ*L berberine or phenol. After 24 h, the media were removed and the cells were washed twice with phosphate buffer solution (PBS). Subsequently, 100 *μ*L CCK-8 working solution was added to each well. After 2 h of incubation at 37°C, the optical density (OD) was read on a microplate reader (Thermo, USA) at a test wavelength of 450 nm against a reference wavelength of 630 nm. The percentages of viability were calculated as follows:

(1) $$\begin{array}{c} \text{Cell viability}\left(\;\text{\%}\;\right)=\left[\;\frac{\left({\text{OD}}_{\text{Sample}}-{\text{OD}}_{\text{Blank}}\right)}{\left({\text{OD}}_{\text{Control}}-{\text{OD}}_{\text{Blank}}\right)}\;\right]\times 100\%. \end{array}$$

### 2.4. Flow Cytometry Analysis of Cell Cycle

To investigate the antiproliferative toxicity of berberine against L929 cells, we evaluated the effect of berberine on progression of the cell cycle. L929 cells in the exponential phase of growth were treated with berberine at the indicated concentrations (0.005, 0.025, and 0.1 mg/mL) for 24 h. 10 *μ*g/mL NVB was used as positive control. Morphological observations of each group were conducted under an inverted (×100) microscope. Subsequently, cell supernatants were collected, and the cells were stained with propidium iodide followed by harvesting. The DNA content of cells was determined with a FACS-*Calibur* flow cytometry and* Mod-Fit* analytic software (Verity Software House. USA).

### 2.5. Examination of DNA Damage by Comet Assay

In this experiment, we employed comet assay to evaluate the genotoxicity induced by berberine on L929 cells with slight modifications according to parameters set by Singh et al. and Gao et al. [13, 14]. Briefly, cells were induced by the indicated concentrations (0.0025, 0.0125, and 0.025 mg/mL) of berberine for 24 h. H_2_O_2_ (50 *μ*M) was used as positive control. After* trypsinization* cell density was adjusted to 2  ×  10^5^  cells/mL. The cell suspension was mixed with 1% LMA at 1 : 3 (v/v) and layered on the surface of special slides (40 *μ*L/well) which were made by the covers of 24-well cell culture plates instead of traditional slides. After slides had gelled at 4°C for 30 min, these slides were immersed in freshly prepared cold lysing solution and refrigerated for 1–1.5 h prior to electrophoresis, despiralization, neutralization, and finally staining. The whole process was carried out in a condition of cold and dimly lit environment in order to minimize additional repair or damage by external variant sources. In essence, constancy was observed. Comet images of each slide were taken by a fluorescence microscope (OLYMPUS IX71, Japan) and analyzed with Comet Assay Software* Pect* (CASP) [15, 16].

### 2.6. Detection of ROS Level

To examine whether ROS was involved in berberine-induced L929 cytotoxicity, a fluorescent dye, DCFH-DA, was utilized prior to flow cytometric analysis. Cells were seeded in 60 mm dishes and cultured at 37°C with 5% CO_2_. Following the treatment with berberine at indicated concentrations for 24 h, cells were dyed with DCFH-DA at 37°C for 20 min.* Rosup* (50 mg/mL) was used as positive control. After being washed with PBS, 10,000 cells were detected with flow cytometry, and* Flow-Jo* 7.6 software was utilized to examine the level of intracellular ROS.

### 2.7. Observation of Mitochondrial Membrane Potential

In this study, changes of mitochondrial membrane potential were detected by JC-1 staining. L929 cells in logarithmic phase were treated by different concentrations of berberine (0.005, 0.025, and 0.1 mg/mL) for 24 h. CCCP (10 mM) was used as positive control. After cultivating for 20 min, each hole was washed twice with JC-1 (×1) buffer at ice bath. Six-well plates were then put in the inverted fluorescence microscope. The red and green fluorescent intensity of mitochondrial membrane potential was tested by* Image-J* analysis software.

### 2.8. Measurement of Cell Apoptosis

To examine whether cell apoptosis was involved in berberine-induced L929 cytotoxicity, two fluorescent dyes* Annexin* V-FITC/PI were utilized prior to flow cytometric analysis. L929 cells in the exponential phase of growth were treated with berberine at the indicated concentrations (0.005, 0.025, and 0.1 mg/mL) for 24 h.* Cisplatin* Injection (50 *μ*M) was used as positive control. Treated cells were washed with PBS and collected by centrifugation after treatment with berberine for 24 h. The cell pellets were stained with* Annexin* V-FITC/PI at room temperature for 15 min. Following incubation, 10,000 cells were analyzed with a flow cytometry and* Flow-Jo* 7.6 software was used to analyze cell apoptosis.

### 2.9. Statistical Analysis

Statistical analyses were performed by SPSS 19.0 for Windows software and evaluated by an associated *t*-test or one-way analysis of variance (ANOVA). Data are presented as mean ± standard deviation (SD). The significant level was detected at *p* < 0.05.

## 3. Results

### 3.1. Effects of Berberine on Cell Viability

**Figure 2:**
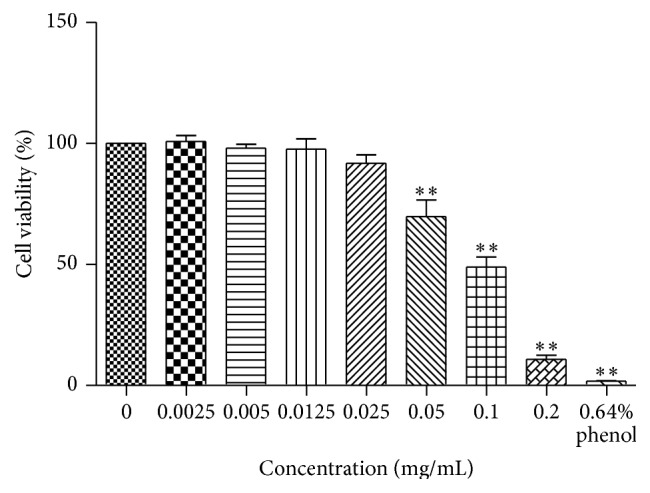
Cell viability of berberine at different concentrations for 24 h was examined against L929 cell lines by CCK-8 assay. The histograms were shown by GraphPad Prism 5.0 (GraphPad Software, Inc., USA). *n* = 4;  ^*∗∗*^ *p* < 0.01 compared with control.

The study investigated the potential cytotoxicity of berberine on L929 by CCK-8 (Figure 2). Berberine did not induce obvious cytotoxicity at doses from 0.0025 mg/mL to 0.025 mg/mL. However, the statistically significant decreases were observed when the berberine concentration was higher than 0.05 mg/mL compared with negative control. Notably, when cells were treated with 0.2 mg/mL berberine, the viability of L929 cells was 10.87 ± 3.41, indicating a significant cytotoxicity (*p* < 0.01).

### 3.2. Effects of Berberine on Cell Morphological Characteristics

**Figure 3:**
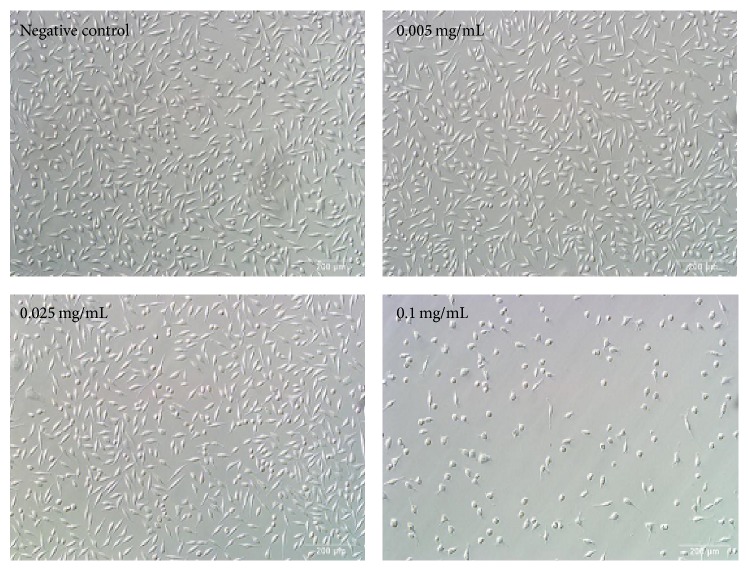
Morphological changes of L929 cells after berberine treatment at different concentrations for 24 h (×100).

To verify berberine-induced cytotoxicity, we examined the changes of cell morphology in L929 cells exposed to 0.005–0.1 mg/mL dosage of berberine for 24 h. Cells in the control group were of high density and of adherent growth, while cells induced by 0.1 mg/mL berberine were shrinking, round, and detached (Figure 3).

### 3.3. Effects of Berberine on Cell Cycle

**Figure 4:**
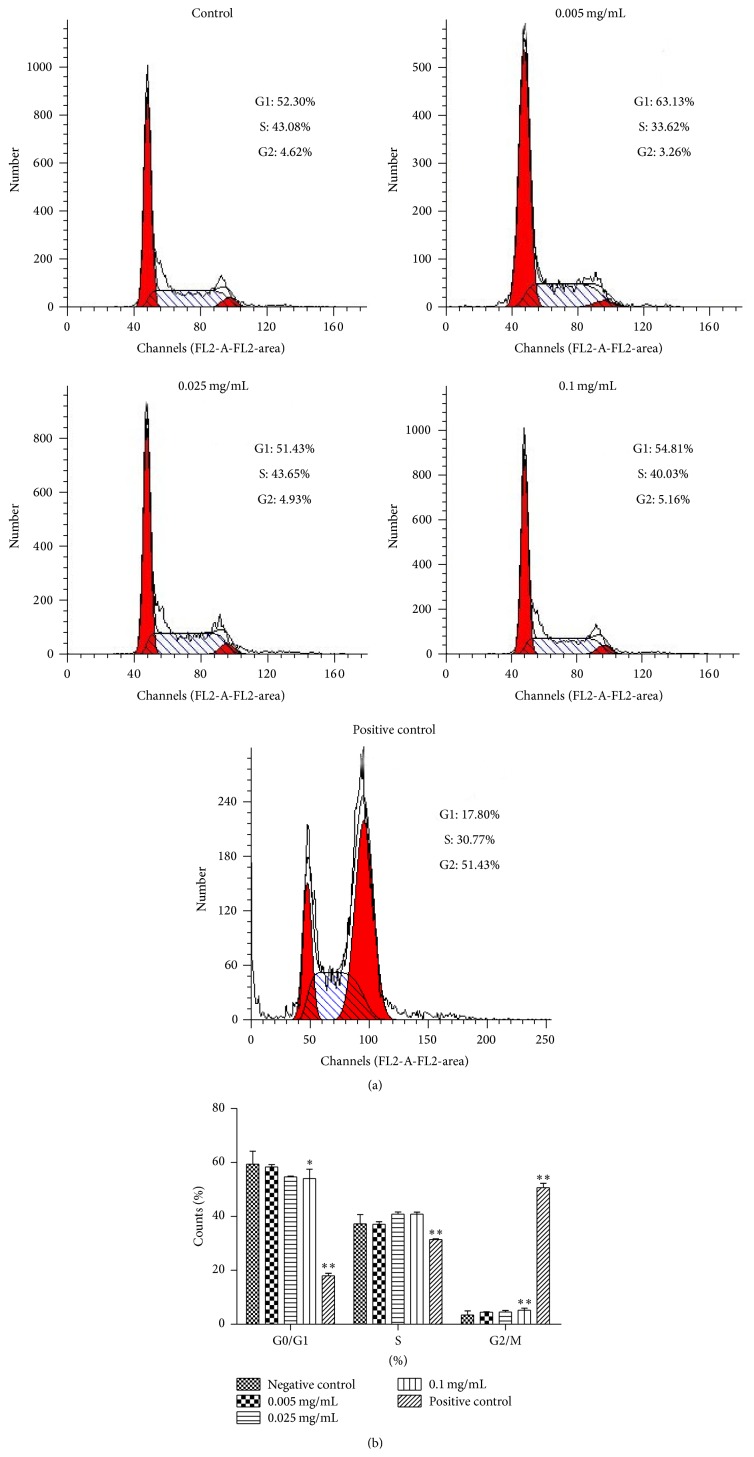
Effects of berberine on L929 cell cycle. (a) The cells were exposed to different berberine concentrations for 24 h. Cell cycle after propidium iodide staining was detected by flow cytometry. (b) The quantities of cell cycle were analyzed. *n* = 3. ^*∗*^ *p* < 0.05 and ^*∗∗*^ *p* < 0.01 compared with the control.

Figure 4 shows the results of cell cycle analysis. A statistically significant increase was demonstrated attributing to the cells proportion in G2/M phase from (3.40 ± 1.57)% to (5.24 ± 0.71)% at 0.1 mg/mL group (*p* < 0.01). The results suggested that berberine prevented cell cycle progression by arresting cell cycle at G2/M phase, leading to disorders of cell division cycle at the concentration of 0.1 mg/mL.

### 3.4. Effects of Berberine on DNA

**Figure 5:**
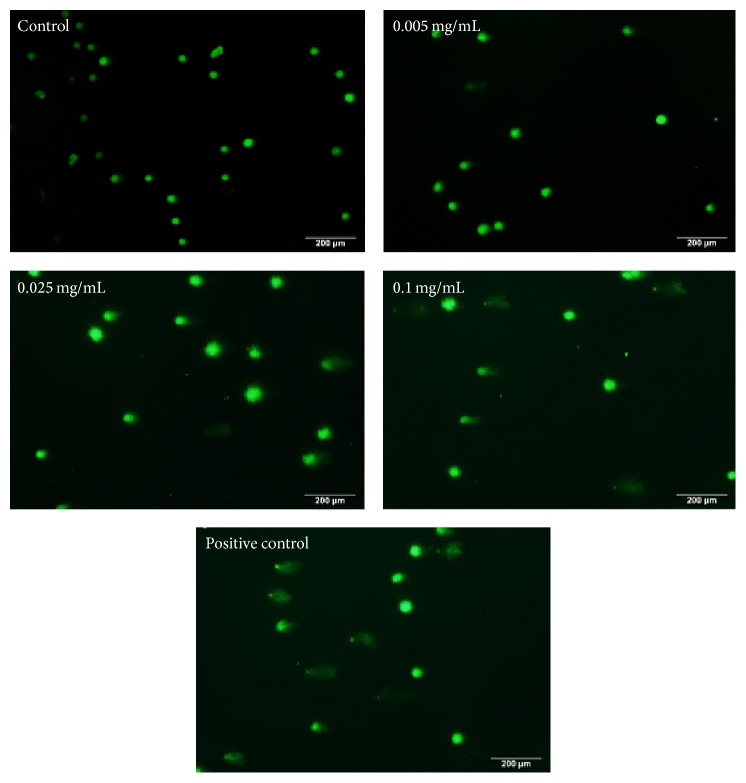
DNA damage of L929 cells stimulated by berberine. The indicated concentrations of berberine were added to cell culture for 24 h. Following dye application with GelGreen, comet images were taken at a magnification (×100) using a fluorescence microscope.

In this study, damage at the single cell level caused by DNA single strand breaks was tested by alkaline comet assay. As shown in Figure 5, only circle fluorescence images could be observed in control. With the increase of the berberine concentration, circular fluorescent anomalies become longer, appearing in comet imagery form. Compared to control, no abnormal DNA changes were observed at 0.0025 mg/mL group; but in 0.0125 mg/mL TL and TDNA% lesion scores were significantly different (*p* < 0.01); in 0.025 mg/mL four injury score indicators were significantly different (*p* < 0.01) (Table 1). The results indicated that the 24 h exposure of L929 cells to berberine increased DNA damage in a dose-dependent manner.

### 3.5. Effects of Berberine on ROS Level

**Figure 6:**
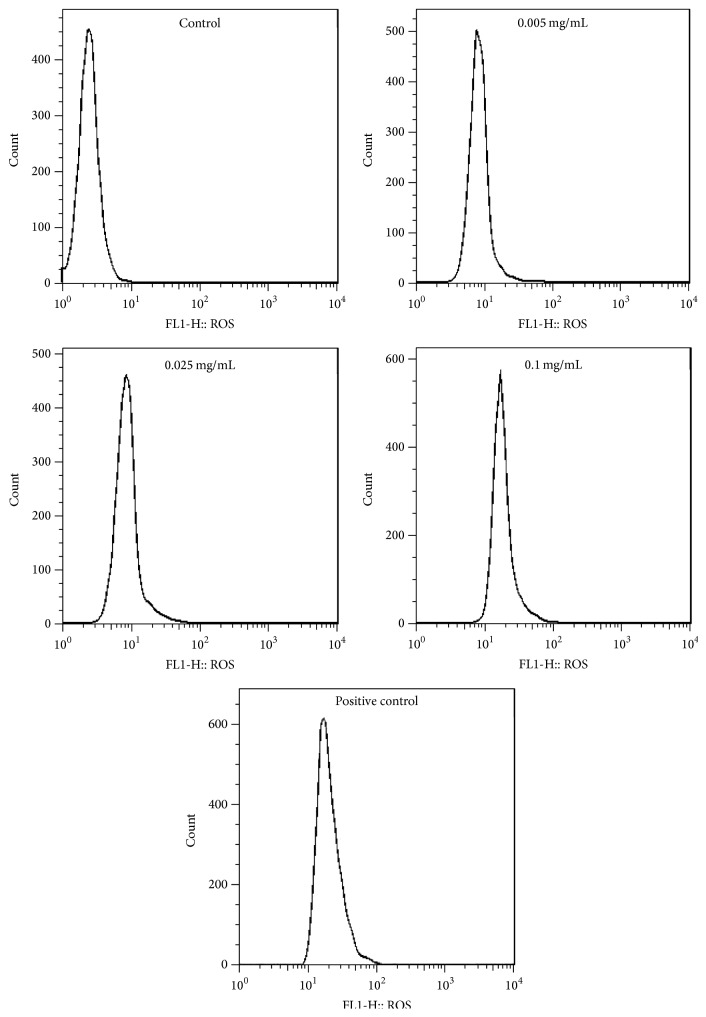
Effect of berberine on L929 intracellular ROS. The cells were exposed to different berberine concentrations for 24 h. ROS level was determined by flow cytometric analysis.

In this test, we found that the intracellular ROS level in treated groups was increasing with statistically significant differences compared with the control (*p* < 0.01) (Figure 6, Table 2). ROS levels of L929 cells treated by berberine concentration in 0.025 mg/mL and 0.05 mg/mL resulted in minor differences, but those in 0.01 mg/mL were almost twice the indicative result of the first two variables. These data demonstrated that ROS in L929 having been induced by berberine increased in a dose-dependent manner. The result indicated that cytotoxicity induced by berberine occurs via a mechanism that intrinsically involves ROS accumulation.

### 3.6. Effects of Berberine on Mitochondrial Membrane Potential

**Figure 7:**
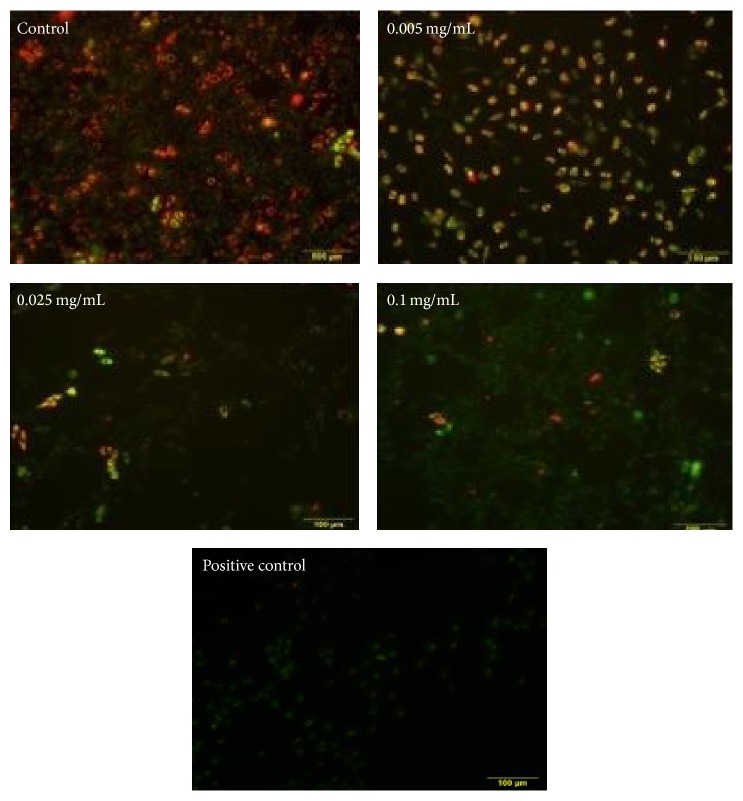
Mitochondrial membrane potential of berberine treated L929 cells after 24 h. Red fluorescence: mitochondrial membrane potential is higher; green fluorescence: mitochondrial membrane potential is lower.

Changes of mitochondrial membrane potential were detected by JC-1 staining, and it showed that there are marked contrasts when compared with the control group. With the concentrations of berberine increasing, red fluorescence intensity eased up, and green fluorescence intensity increased (Figure 7). Accordingly, indicative results display red and green fluorescence ratios as having decreased significantly (*p* < 0.01) (Table 3). The results showed that after 24 h berberine treated L929 cells can display changes within mitochondrial membrane potential efficacy from applicative dose-dependent manner.

### 3.7. Effects of Berberine on Cell Apoptosis

**Figure 8:**
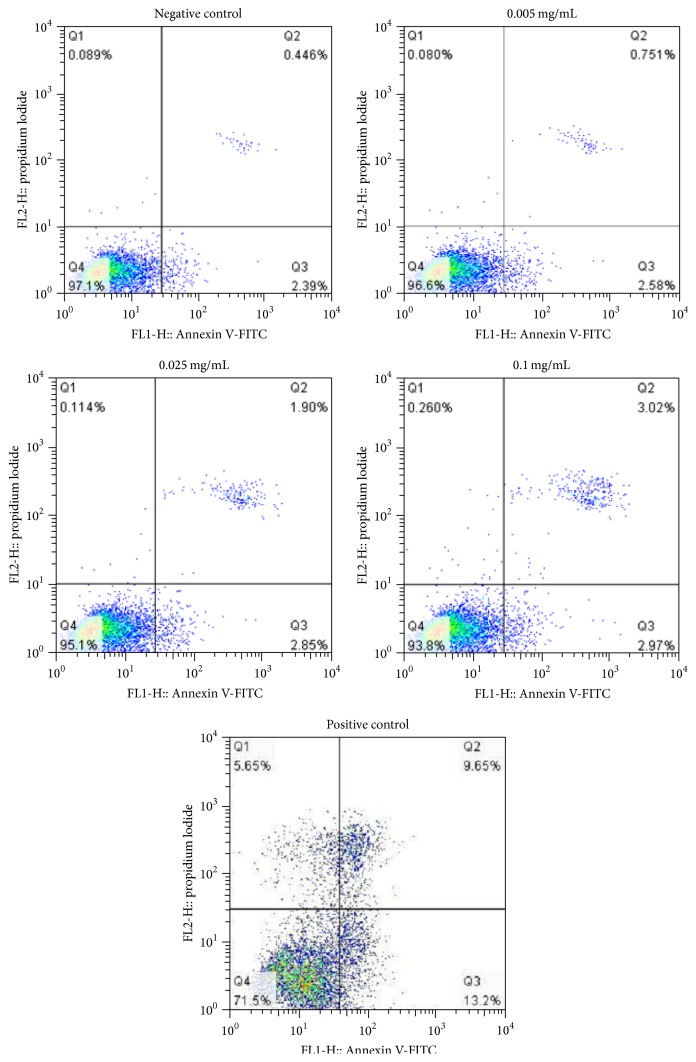
Effects of berberine on L929 cell apoptosis rate. The cells were exposed to different berberine concentrations for 24 h. Cell apoptosis rate after* Annexin* V-FITC/PI staining was detected by a flow cytometry.

The result showed that the 0.1 mg/mL berberine induced almost twofold increase in the apoptosis rate in contrast to the control state (Figure 8). Compared with the control group, with the increase of concentrated berberine, the total apoptosis rate of the cells increased (*p* < 0.01) (Table 4), signifying dose-dependent variance. Indicatively, most of the cells belonging to the Q2 quadrant signify berberine can induce L929 cell apoptosis, factored as late apoptosis trending (Figure 8) following 24 h treatment conditions. These results indicate that cell apoptosis was resultant from berberine-induced L929 cell cytotoxicity.

## 4. Analytical Discussion

With the continual discovery and application of new pharmacological active substances, there have been increasing concerns about safety and potential toxicity of berberine preparations. Clinical results have been causative leading to the banned or controlled use of berberine as a result of various negative observations displaying adverse side effects. The security issues surrounding the applicative use of berberine have attracted attention internationally which has resulted in conditional status from observations drawn by methodical research. Previous clinical reports indicate intravenous injection of berberine contributes to dilation of blood vessels and consequent lower blood pressure [17] and inhibition of the function of heart [18] or in the extremity it can even result in fatality of the subject. Berberine use can also result in malabsorption of vitamin B, inducing peripheral neuritis [19]. In addition, potential genotoxicity, such as gene mutation in yeast, has also been reported [20]. In practicality, berberine appears to have a wide therapeutic index medium demonstrated by the variable range (up to 0.5 g) of doses which are used in clinical application without apparent side effects. However, if used indiscriminately and inappropriately, then it may give rise to unexpected adverse effects [21]. Based on these clinical reports, many scholars have carried out a series of experiments to verify these toxicities. Interestingly, they indeed found that almost all of these adverse reactions were due to misuse or abuse, especially those pertaining to excessive dosage. For example, Ma et al. demonstrated that berberine held the maximum cytotoxicity in four main alkaloids of* Rhizoma Coptidis* and the cytotoxicity trended from a dose- and time-dependent applicative manner [22]; Jahnke et al. believed that berberine at certain levels leads to developmental toxicity both in rats and mice and pointed out that the lowest-observed adverse effect level (LOAEL) was 7250 ppm and 14500 ppm, respectively [23]. As a generally accepted observance, human tolerance range of berberine prior to toxicity is less than 0.5 g, with an overdose of berberine (more than 4 g) potentially resulting in nausea, diarrhea, allergies, and other observed and adversative reactions. Yi et al. demonstrated that the currently recommended highest dose of berberine is relatively safe, as it did not cause deaths or any abnormal changes in subchronic toxicity tests on rats [24]. Assumptions drawn from testing determine that it is necessary to regulate berberine dosage according to indicative safe levels to ensure security and continuity of its use.

Many researchers, both domestically and globally, have suggested* in vitro* cytotoxicity was demonstrated in direct correlation to the toxicity findings from clinical research conducted* in vivo* [25–27]. The reference index of cytotoxicity LD50 was recommended by the National Toxicology Group Program and the Toxicology of the Experimental Evaluation Center (NICEATM). The report said the value of LD50 used in assays could obtain more accurate results of the acute toxicity of compounds* in vivo*. The result of a preliminary study of cytotoxicity of* Rhizoma Coptis* and berberine on four kinds of cells (GES-1 cell, BRL cell, NRK cell, and L929 cell) cultured* in vitro* was performed by our study team under the above aforementioned toxicology testing standards. Furthermore,* in vitro* evaluation methods are adopted at early screening stages for new chemical substances to be placed on registry for commercial exploitation as an approved drug. The* in vitro* standard testing procedure can save time, effort, and money and has been fortified procedurally as an accepted standard by many scholars [28–30]. Although the safety of berberine at certain dosage is questioned (as described above), until now its cytotoxicity was not extensively documented on all parameters other than detecting the cell survival rate with MTT assay [22]. This study was conducted to build on the case of the cytotoxic mechanics of berberine. The chosen international standard (UB/T16886.5-2003) of Test Cell L929 was employed as a common biological evaluation of medical systems practices, with the goal of determining berberine cytotoxicity presence, form, and result at different metrics (cell survival rate, cell cycle, ROS, DNA damage, etc.). In reference to the stable property of berberine throughout the assay, the following constants were observed: explored in the condition of light 5 d, its content from 99.81% to 99.44%; at 80°C 5 d, its content from 99.81% to 99.14%; at wetness 92.5% 5 d, its content from 99.81% to 99.36% [31]. We chose 24 hours of treatment under the condition of* in vitro* culture, which is adopted widely by related research.

CCK-8 analysis is cleaved to formazan by cellular dehydrogenases. The amount of dye converted is directly proportional to the number of living cells [32, 33], clearly defining a direct reflection of cytotoxicity. Observances of berberine dosage higher than 0.05 mg/mL showed a significant lethal effect on L929 cells, indicating that berberine at a certain concentration has detrimentally evident cytotoxic status. From findings, conclusions are drawn that, within a reasonable dosage range, the application of berberine can be considered safe and effective. The normal process of cell proliferation is accomplished by an orderly cell division cycle, where precise regulation of the cell cycle is essential for orderly proliferation. The conversion of the cell cycle relies on the activation and expression of a series of cyclins (e.g., cyclins B, D, and E) and cyclin dependent kinases (e.g., CDK 2, 4, and 6) [34]. Flow cytometry analysis showed that berberine at 0.1 mg/mL could induce G2/M phase arrest. Meanwhile this concentration tested on cell viability has shown remarkably significant inhibitive stimuli on normal cyclical functioning of cells. Previous studies have shown that if the increase of cells in G2/M phase is not caused by the result of promoted proliferation, it may be related to DNA damage or other changes. Indeed, our result was compatible with the latter findings, adding further weight to that reported observance. Cell cycle checkpoints were actively observed and recorded and cell cycle arrest resulted from protective cellular reaction leading to cell cycle arrest [35, 36]. The result suggested that berberine cytotoxicity of L929 was partially ascribed to cell cycle arrest, although this phenomenon may not be the most pivotal factor in the study.

An important element of this study, with respect to cell apoptosis, focused more specifically on DNA damage-induced apoptosis. The accumulation of ROS can induce the increase of free radicals, which can easily penetrate and diffuse into the nucleus, directly acting on nucleic acid, causing the base modification and DNA strand breaks, resulting in DNA damage [37–39]. DNA strand breaks can activate DNA-dependent protein kinase (DNA-PK), which can phosphorylate and activate P53, which in turn promoted the expression of downstream proapoptotic genes (such as FasL/Fas, Apaf-l, Bax, and DR5) and thus initiated apoptosis [40–42]. DNA damage can cause distortion or mutation [34]. Alkaline comet assay (pH > 13) is considered to detect DNA damage such as single-strand breaks (SSBs), double-strand breaks (DSBs), alkali labile sites (ALS), and incomplete excision repair sites. This method is sensitive and intuitionistic [43]. At the same time, in view of the fluorescence intensity between the cell cycle correlations [44], we utilized custom modified alkaline comet assay techniques to detect single-strand breaks (SSBs) in DNA based damage. Consistent with this report, our results show that berberine induced DNA damage in L929 cells, and four kinds of analytical parameters achieved a satisfactory consistency. Interestingly, we observed no significant differences in cell viability at 0.025 mg/mL but found obvious DNA damage in the comet assay at the same concentration. Therefore, we speculate that damage of genetic material is prior to biochemical changes in cells. This damage partially accounted for a more potent cytotoxicity of berberine on L929 cells.

Reactive oxygen species (ROS) is of great significance in cell apoptosis [45]. The study showed that ROS were mainly derived from the mitochondrial oxidative transport chain (Mito-ETC), which was completed by NADH dehydrogenase (complex I) and cytochrome C reductase (complex III). In the process of oxidative metabolism, oxygen molecules formed a superoxide anion by reducing an electron (initial ROS formation), superoxide anion generated H_2_O_2_ by superoxide dismutase (SOD) within the mitochondria or cytoplasm, in turn producing high activity of ^∙^OH through oxidation and/or Haber Weiss reaction with the presence of metal ions, resulting in the decrease of mitochondrial transmembrane potential, mitochondrial release of cytochrome C, and the intracellular proteins, lipids, and DNA form oxidative damage and thus induced cell apoptosis [46–50]. In addition, the external factors including damage, radiation, chemotherapy drugs, and other stimuli might cause oxidation/antioxidant imbalance, which can be potentially triggering the apoptotic signal transduction pathway. After the initiation of apoptosis, ROS may further accelerate the process of apoptosis [51]. The accumulated ROS can cause severe damage to macromolecular substances, especially nucleic acid, which can cause DNA strand breaks. DNA damage can activate p53 signaling pathway leading to mitochondrial dysfunction and trigger factors leading to apoptosis (oxidative damage) by activating caspase [52]. Our results show that berberine concentration in 0.005–0.1 mg/mL can cause the accumulation of ROS, eventually leading to cell toxicity.

Apoptosis is programmed cell death which results as interaction from a variety of inducements. However, mitochondrial pathway is the classical pathway of apoptosis, and the mitochondria contain a variety of apoptosis related factors. When the mitochondrial membrane is damaged, the mitochondrial outer membrane permeability increases, the mitochondrial membrane potential reduces, and it releases a variety of proapoptotic factors. Therefore, mitochondria play an important role in apoptosis, appropriately allied as a combustor of apoptosis phenomenon [53]. The result of JC-1 staining showed berberine can significantly reduce mitochondrial membrane potential. Our test showed typical morphological features of apoptosis such as cell density decrease and shrinking and observed spherical features. The data also confirmed that berberine could induce L929 cell apoptosis in a dose-dependent manner. These results pointed out that berberine-induced apoptosis was relevant with the proliferation inhibition as well as cytotoxic effect.

In summary, the advantages of this study lie in the fact that the experiments were performed according to standard procedures using relatively simple, well-established, and routine methods. Moreover, previous studies of berberine cytotoxicity in cell cycle, ROS, DNA damage, and apoptosis are to date not conclusively or intensively reported in studies conducted by other groups. In the case study to determine the cytotoxic mechanisms of berberine in a clear more concise manner, the authors chose the international standard (UB/T16886.5-2003) of L929 cells as a test cell for examining several different levels of cytotoxicity. However, there still exists scope to expound on these findings as it is noted that L929 is not a comprehensively targeted cell. If a deep research based on the results of this study will be carried out, a specific target cell model should be selected based on experimental purposes (such as genetic toxicity and cell cycle progression). Further studies are needed to elucidate the complicated mechanisms of berberine cytotoxicity, thereby guiding the clinical applications in a secure and clinically sound manner.

## Figures and Tables

**Table 1:** Effect of berberine on DNA of L929 cells (*n* = 50).

Concentration (mg/mL)	Indicators			
	TL (*μ*m)	TDNA (%)	TM (*μ*m)	Olive TM (*μ*m)
0	3.25 ± 0.46	0.37 ± 0.25	0.01 ± 0.01	0.18 ± 0.13
0.0025	8.67 ± 1.80	0.27 ± 0.20	0.02 ± 0.01	0.19 ± 0.11
0.0125	133.4 ± 18.50^*∗∗*^	11.73 ± 6.13^*∗∗*^	7.11 ± 3.76	6.14 ± 1.96
0.0250	240.1 ± 27.21^*∗∗*^	26.67 ± 10.55^*∗∗*^	35.97 ± 15.26^*∗∗*^	24.50 ± 9.31^*∗∗*^
Positive control	286.3 ± 53.71^*∗∗*^	84.12 ± 9.95^*∗∗*^	243.5 ± 64.27^*∗∗*^	131.88 ± 37.61^*∗∗*^

Visual scorings of DNA damage were based on the fluorescence intensity. TL: tail length; TDNA: tail DNA%; TM: tail moment; OTM: olive tail moment. Values are expressed as mean ± SD. ^*∗∗*^ *p* < 0.01 compared with the control.

**Table 2:** Effect of berberine on intracellular ROS of L929 cells (*n* = 3).

Concentration (mg/mL)	ROS
Control	2.68 ± 0.08
0.005	9.21 ± 1.27^*∗∗*^
0.025	10.64 ± 1.19^*∗∗*^
0.1	21.40 ± 1.59^*∗∗*^
Positive control	56.23 ± 1.07^*∗∗*^

Values are expressed as mean ± SD. Compared with control, ^*∗∗*^ *p* < 0.01.

**Table 3:** Mitochondrial membrane potential of berberine treated L929 cells (*n* = 3).

Concentration (mg/mL)	The ratio of green and red fluorescence
Control	1.332 ± 0.045
0.005	1.420 ± 0.042^*∗∗*^
0.025	1.088 ± 0.037^*∗∗*^
0.1	0.801 ± 0.042^*∗∗*^
Positive control	0.477 ± 0.033^*∗∗*^

Values are expressed as mean ± SD. Compared with control, ^*∗∗*^ *p* < 0.01.

**Table 4:** Total apoptosis rate of berberine treated L929 cells (*n* = 3).

Concentration (mg/mL)	Total apoptosis rate
Control	2.835 ± 0.004
0.005	3.784 ± 0.045^*∗∗*^
0.025	4.790 ± 0.040^*∗∗*^
0.1	5.554 ± 0.021^*∗∗*^
Positive control	22.97 ± 0.032^*∗∗*^

Values are expressed as mean ± SD. Compared with control, ^*∗∗*^ *p* < 0.01.
